# 6-[(Dimethyl­amino)methyl­ene­amino]-1,3-dimethyl­pyrimidine-2,4(1*H*,3*H*)-dione dihydrate

**DOI:** 10.1107/S1600536808024021

**Published:** 2008-08-06

**Authors:** Subrata Das, Binoy K. Saikia, B. Sridhar, Ashim J. Thakur

**Affiliations:** a Department of Chemical Sciences, Tezpur University, Tezpur 784 028, India; b Laboratory of X-ray Crystallography, Indian Institute of Chemical Technology, Hyderabad 500 607, India

## Abstract

Uracil, the pyrimidine nucleobase, which combined with adenine forms one of the major motifs present in the biopolymer RNA, is also involved in the self-assembly of RNA. In the title compound, C_9_H_14_N_4_O_2_·2H_2_O, the asymmetric unit contains one dimethyl­amino­uracil group and two water mol­ecules. The plane of the N=C—NMe_2_ side chain is inclined at 27.6 (5)° to the plane of the uracil ring. Both water mol­ecules form O—H⋯O hydrogen bonds with the carbonyl O atoms of the uracil group. Additional water–water hydrogen-bond inter­actions are also observed in the crystal structure. The O—H⋯O hydrogen bonds lead to the formation of a two-dimensional hydrogen-bonded network cage consisting of two dimethyl­amino­uracil groups and six water mol­ecules.

## Related literature

For related literature, see: Pontikis & Monneret (1994 ▶); Sasaki *et al.* (1998 ▶); Sivakova & Rowan (2005 ▶); Thakur *et al.* (2001 ▶).
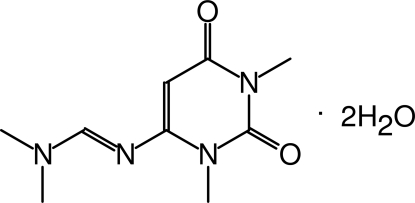


## Experimental

### 

#### Crystal data

C_9_H_14_N_4_O_2_·2H_2_O
*M* *_r_* = 246.27
Triclinic, 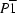

*a* = 7.1310 (5) Å
*b* = 9.8571 (7) Å
*c* = 9.9160 (7) Å
α = 92.921 (1)°
β = 101.916 (1)°
γ = 109.912 (1)°
*V* = 635.62 (8) Å^3^
*Z* = 2
Mo *K*α radiation
μ = 0.10 mm^−1^
*T* = 294 (2) K
0.23 × 0.17 × 0.12 mm

#### Data collection

Bruker SMART APEX CCD area-detector diffractometer
Absorption correction: none
6112 measured reflections
2231 independent reflections
2017 reflections with *I* > 2˘*I*)
*R*_int_ = 0.019

#### Refinement

*R*[*F* ^2^ > 2σ(*F* ^2^)] = 0.051
*wR*(*F* ^2^) = 0.155
*S* = 1.07
2231 reflections
175 parameters
4 restraints
H atoms treated by a mixture of independent and constrained refinement
Δρ_max_ = 0.34 e Å^−3^
Δρ_min_ = −0.21 e Å^−3^

### 

Data collection: *SMART* (Bruker, 2001 ▶); cell refinement: *SAINT* (Bruker, 2001 ▶); data reduction: *SAINT*; program(s) used to solve structure: *SHELXS97* (Sheldrick, 2008 ▶); program(s) used to refine structure: *SHELXL97* (Sheldrick, 2008 ▶); molecular graphics: *SHELXTL/PC* (Sheldrick, 2008 ▶) and *PLATON* (Spek, 2003 ▶); software used to prepare material for publication: *SHELXL97*.

## Supplementary Material

Crystal structure: contains datablocks I, global. DOI: 10.1107/S1600536808024021/er2056sup1.cif

Structure factors: contains datablocks I. DOI: 10.1107/S1600536808024021/er2056Isup2.hkl

Additional supplementary materials:  crystallographic information; 3D view; checkCIF report

## Figures and Tables

**Table 1:** Hydrogen-bond geometry (Å, °)

*D*—H⋯*A*	*D*—H	H⋯*A*	*D*⋯*A*	*D*—H⋯*A*
O1*W*—H1*W*⋯O2	0.86 (1)	1.93 (1)	2.784 (2)	173 (3)
O1*W*—H2*W*⋯O1*W*^i^	0.86 (7)	2.01 (4)	2.771 (4)	147 (6)
O2*W*—H3*W*⋯O1	0.85 (1)	2.01 (2)	2.808 (2)	157 (3)
O2*W*—H4*W*⋯O1*W*^ii^	0.86 (3)	1.95 (2)	2.777 (3)	163 (6)

Symmetry codes: (i) 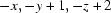; (ii) 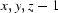.

**Fig. 1.**
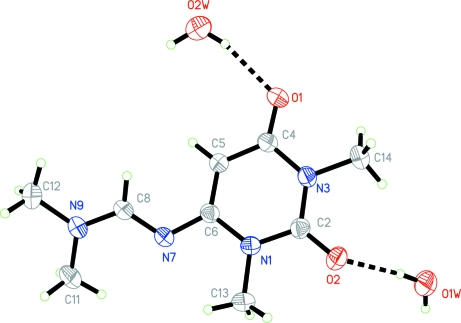
A view of the (I), with the atom-numbering scheme. Displacement ellipsoids are drawn at the 30% probability level and H atoms are shown as small spheres of arbitrary radii. Dashed lines indicates hydrogen bonds.

**Fig. 2.**
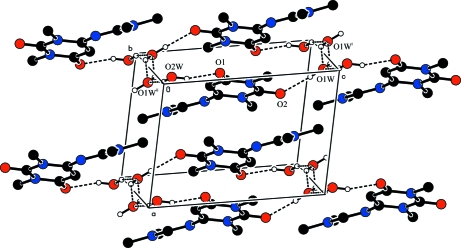
A packing diagram for (I), viewed down the b axis. H atoms not involved in hydrogen bonding have been omitted for clarity. [Symmetry code:(i) -x, -y + 1, -z + 2; (ii) x, y, z - 1].

## supplementary crystallographic information

### Comment

Uracil, the pyrimidine nucleobase, combined with Adenine comprises one of the major motifs present in the biopolymer RNA, is also involved in the self-assembly of RNA(Sivakova & Rowan, 2005) The versatility of uracil and its derivatives, particularly the annulated one, is well recognized by synthetic (Sasaki *et al.*, 1998) as well as biological chemists (Pontikis & Monneret, 1994) owing to their wide range of biological activities. The chemistry of uracil moiety and its derivatives have expanded enormously in the past decades only because of its mechanistic, synthetic and biological importance which made them of substantial experimental and theoretical interest.

Synthesis and characterization of the title compound (I) was reported recently from our laboratory (Thakur *et al.*, 2001), through the reaction of 6–amino–1,3–dimethylbarbituric acid with (DMF–DMA) under thermal condition or Microwave irradiation in the solid state. Our ongoing present research program is aimed at synthesizing fused pyrimidine derivatives of biological significances. Also we have been investigating the rotational barrier of the two methyl groups in the exocyclic N9-Me_2_ part in (I), which will help us in understanding the mechanism of the Diels Alder reaction of (I).

The asymmetric unit of (I), comprises one dimethylamino uracil moiety and two water molecules (Fig. 1). The six-membered uracil ring is planar and the plane of its attached side chain is inclined 27.6 (5)° to the plane of the uracil ring. The torsion (C6-N7-C8-C9) = 174.4 (2)°.

The crystal structure is stabilized by O—H···O hydrogen bonds (Table 1). Both the water (O1W and O2W) molecules form O—H···O hydrogen bonds with the carbonyl (O1 and O2) atoms of the uracil moiety. In addition, water···water interactions are also observed in the crystal structure. The water molecules interconnect each other and in turn links the uracil moiety, thereby forming a two-dimensional hydrogen-bonded network cage consists of two dimethylamino uracil moieties and six water molecules (Fig.2).

### Experimental

In order to obtain suitable single crystals for this study, the title compound was dissolved in ethanol (98%) and the solution was allowed to evaporate very slowly.

### Refinement

The H atoms of the water molecules were located in a difference Fourier map and refined isotropically. Distance restraints were also applied to the H atoms of the water molecules with a set value of 0.86 (1) Å. All other H atoms were positioned geometrically and treated as riding on their parent C atoms, with C—H distances of 0.93 - 0.96 Å, and with *U*_iso_(H) values of 1.5*U*_eq_(C) for methyl H atoms and 1.2*U*_eq_(C) for the other H atoms. The methyl groups were allowed to rotate but not to tip.

### Crystal data

**Table:**

C_9_H_14_N_4_O_2_·2H_2_O	*Z* = 2
*M**_r_* = 246.27	*F*_000_ = 264
Triclinic, *P*1	*D*_x_ = 1.287 Mg m^−^^3^
Hall symbol: -P 1	Mo *K*α radiation λ = 0.71073 Å
*a* = 7.1310 (5) Å	Cell parameters from 3875 reflections
*b* = 9.8571 (7) Å	θ = 2.4–27.9º
*c* = 9.9160 (7) Å	µ = 0.10 mm^−^^1^
α = 92.921 (1)º	*T* = 294 (2) K
β = 101.916 (1)º	Block, colorless
γ = 109.912 (1)º	0.23 × 0.17 × 0.12 mm
*V* = 635.62 (8) Å^3^	

### Data collection

**Table:**

Bruker SMART APEX CCD area-detector diffractometer	2017 reflections with *I* > 2˘*I*)
Radiation source: fine-focus sealed tube	*R*_int_ = 0.019
Monochromator: graphite	θ_max_ = 25.0º
*T* = 294(2) K	θ_min_ = 2.1º
ω scans	*h* = −8→8
Absorption correction: none	*k* = −11→11
6112 measured reflections	*l* = −11→11
2231 independent reflections	

### Refinement

**Table:**

Refinement on *F*^2^	Secondary atom site location: difference Fourier map
Least-squares matrix: full	Hydrogen site location: inferred from neighbouring sites
*R*[*F*^2^ > 2σ(*F*^2^)] = 0.051	H atoms treated by a mixture of independent and constrained refinement
*wR*(*F*^2^) = 0.155	*w* = 1/[σ^2^(*F*_o_^2^) + (0.0891*P*)^2^ + 0.1375*P*] where *P* = (*F*_o_^2^ + 2*F*_c_^2^)/3
*S* = 1.07	(Δ/σ)_max_ = 0.001
2231 reflections	Δρ_max_ = 0.34 e Å^−^^3^
175 parameters	Δρ_min_ = −0.21 e Å^−^^3^
4 restraints	Extinction correction: SHELXL97 (Sheldrick, 2008), Fc^*^=kFc[1+0.001xFc^2^λ^3^/sin(2θ)]^-1/4^
Primary atom site location: structure-invariant direct methods	Extinction coefficient: 0.063 (11)

### Special details

**Table:**

Geometry. All e.s.d.'s (except the e.s.d. in the dihedral angle between two l.s. planes) are estimated using the full covariance matrix. The cell e.s.d.'s are taken into account individually in the estimation of e.s.d.'s in distances, angles and torsion angles; correlations between e.s.d.'s in cell parameters are only used when they are defined by crystal symmetry. An approximate (isotropic) treatment of cell e.s.d.'s is used for estimating e.s.d.'s involving l.s. planes.
Refinement. Refinement of *F*^2^ against ALL reflections. The weighted *R*-factor *wR* and goodness of fit *S* are based on *F*^2^, conventional *R*-factors *R* are based on *F*, with *F* set to zero for negative *F*^2^. The threshold expression of *F*^2^ > σ(*F*^2^) is used only for calculating *R*-factors(gt) *etc*. and is not relevant to the choice of reflections for refinement. *R*-factors based on *F*^2^ are statistically about twice as large as those based on *F*, and *R*- factors based on ALL data will be even larger.

### Fractional atomic coordinates and isotropic or equivalent isotropic displacement parameters (Å^2^)

**Table:**

	*x*	*y*	*z*	*U*_iso_*/*U*_eq_	
C2	0.2362 (3)	0.44519 (18)	0.63139 (17)	0.0495 (4)	
C4	0.0848 (3)	0.29150 (17)	0.40673 (18)	0.0509 (4)	
C5	0.1553 (3)	0.41767 (17)	0.34303 (17)	0.0513 (4)	
H5	0.1232	0.4100	0.2465	0.062*	
C6	0.2691 (2)	0.55089 (16)	0.41829 (17)	0.0459 (4)	
C8	0.3784 (2)	0.67157 (17)	0.24338 (18)	0.0508 (4)	
H8	0.3574	0.5808	0.1980	0.061*	
C11	0.4645 (4)	0.9307 (2)	0.2396 (3)	0.0780 (6)	
H11A	0.3631	0.9631	0.1863	0.117*	
H11B	0.5992	0.9970	0.2397	0.117*	
H11C	0.4489	0.9274	0.3334	0.117*	
C12	0.4759 (4)	0.7733 (3)	0.0408 (2)	0.0826 (7)	
H12A	0.4610	0.6745	0.0139	0.124*	
H12B	0.6128	0.8368	0.0423	0.124*	
H12C	0.3791	0.7997	−0.0247	0.124*	
C13	0.4247 (3)	0.70386 (19)	0.64897 (19)	0.0633 (5)	
H13A	0.3316	0.7448	0.6760	0.095*	
H13B	0.5070	0.7687	0.5970	0.095*	
H13C	0.5123	0.6902	0.7305	0.095*	
C14	0.0539 (3)	0.1852 (2)	0.6239 (2)	0.0682 (5)	
H14A	0.1497	0.1355	0.6337	0.102*	
H14B	−0.0781	0.1206	0.5708	0.102*	
H14C	0.0426	0.2164	0.7142	0.102*	
N1	0.3072 (2)	0.56267 (14)	0.56224 (14)	0.0491 (4)	
N3	0.1267 (2)	0.31263 (14)	0.55177 (15)	0.0515 (4)	
N7	0.3477 (2)	0.67916 (14)	0.36771 (15)	0.0520 (4)	
N9	0.4379 (2)	0.78672 (15)	0.17847 (16)	0.0601 (4)	
O1	−0.0098 (2)	0.16688 (13)	0.34493 (14)	0.0693 (4)	
O2	0.2732 (2)	0.45919 (15)	0.75861 (13)	0.0659 (4)	
O1W	0.0831 (3)	0.39489 (19)	0.97812 (18)	0.0879 (5)	
H1W	0.142 (4)	0.408 (3)	0.910 (2)	0.107 (9)*	
H2W	0.063 (13)	0.462 (7)	1.025 (6)	0.29 (4)*	
O2W	−0.0201 (5)	0.1195 (2)	0.0614 (2)	0.1259 (9)	
H3W	0.002 (6)	0.117 (4)	0.1489 (12)	0.137 (13)*	
H4W	0.035 (9)	0.202 (3)	0.035 (6)	0.27 (3)*	

### Atomic displacement parameters (Å^2^)

**Table:**

	*U*^11^	*U*^22^	*U*^33^	*U*^12^	*U*^13^	*U*^23^
C2	0.0571 (9)	0.0503 (9)	0.0474 (9)	0.0243 (7)	0.0172 (7)	0.0084 (7)
C4	0.0550 (9)	0.0433 (9)	0.0534 (10)	0.0150 (7)	0.0150 (7)	0.0052 (7)
C5	0.0591 (10)	0.0438 (9)	0.0457 (9)	0.0122 (7)	0.0123 (7)	0.0056 (7)
C6	0.0500 (8)	0.0412 (8)	0.0497 (9)	0.0182 (7)	0.0154 (7)	0.0067 (6)
C8	0.0543 (9)	0.0401 (8)	0.0540 (9)	0.0115 (7)	0.0134 (7)	0.0075 (7)
C11	0.0987 (16)	0.0435 (10)	0.0887 (15)	0.0145 (10)	0.0328 (12)	0.0165 (9)
C12	0.1047 (17)	0.0728 (13)	0.0695 (13)	0.0182 (12)	0.0398 (12)	0.0212 (10)
C13	0.0772 (12)	0.0492 (10)	0.0566 (11)	0.0160 (9)	0.0158 (9)	−0.0056 (8)
C14	0.0869 (13)	0.0527 (10)	0.0671 (12)	0.0213 (9)	0.0260 (10)	0.0223 (9)
N1	0.0591 (8)	0.0421 (7)	0.0469 (8)	0.0178 (6)	0.0158 (6)	0.0026 (6)
N3	0.0620 (8)	0.0434 (7)	0.0532 (8)	0.0191 (6)	0.0203 (6)	0.0126 (6)
N7	0.0604 (8)	0.0392 (7)	0.0539 (8)	0.0132 (6)	0.0163 (6)	0.0073 (6)
N9	0.0686 (9)	0.0455 (8)	0.0609 (9)	0.0098 (7)	0.0210 (7)	0.0130 (6)
O1	0.0873 (9)	0.0408 (7)	0.0646 (8)	0.0042 (6)	0.0191 (7)	0.0023 (5)
O2	0.0870 (9)	0.0680 (8)	0.0465 (7)	0.0292 (7)	0.0213 (6)	0.0099 (6)
O1W	0.1212 (14)	0.0829 (11)	0.0666 (10)	0.0319 (10)	0.0444 (9)	0.0144 (8)
O2W	0.226 (3)	0.0825 (13)	0.0787 (13)	0.0549 (15)	0.0575 (15)	0.0108 (10)

### Geometric parameters (Å, °)

**Table:**

C2—O2	1.225 (2)	C12—N9	1.454 (3)
C2—N3	1.373 (2)	C12—H12A	0.9600
C2—N1	1.376 (2)	C12—H12B	0.9600
C4—O1	1.236 (2)	C12—H12C	0.9600
C4—N3	1.397 (2)	C13—N1	1.471 (2)
C4—C5	1.408 (2)	C13—H13A	0.9600
C5—C6	1.365 (2)	C13—H13B	0.9600
C5—H5	0.9300	C13—H13C	0.9600
C6—N7	1.365 (2)	C14—N3	1.469 (2)
C6—N1	1.388 (2)	C14—H14A	0.9600
C8—N7	1.299 (2)	C14—H14B	0.9600
C8—N9	1.320 (2)	C14—H14C	0.9600
C8—H8	0.9300	O1W—H1W	0.86 (1)
C11—N9	1.449 (3)	O1W—H2W	0.86 (7)
C11—H11A	0.9600	O2W—H3W	0.85 (1)
C11—H11B	0.9600	O2W—H4W	0.86 (3)
C11—H11C	0.9600		
			
O2—C2—N3	122.04 (16)	H12B—C12—H12C	109.5
O2—C2—N1	120.84 (16)	N1—C13—H13A	109.5
N3—C2—N1	117.11 (14)	N1—C13—H13B	109.5
O1—C4—N3	118.78 (15)	H13A—C13—H13B	109.5
O1—C4—C5	125.42 (16)	N1—C13—H13C	109.5
N3—C4—C5	115.80 (14)	H13A—C13—H13C	109.5
C6—C5—C4	122.19 (16)	H13B—C13—H13C	109.5
C6—C5—H5	118.9	N3—C14—H14A	109.5
C4—C5—H5	118.9	N3—C14—H14B	109.5
N7—C6—C5	127.10 (15)	H14A—C14—H14B	109.5
N7—C6—N1	114.39 (14)	N3—C14—H14C	109.5
C5—C6—N1	118.48 (15)	H14A—C14—H14C	109.5
N7—C8—N9	123.01 (16)	H14B—C14—H14C	109.5
N7—C8—H8	118.5	C2—N1—C6	122.46 (14)
N9—C8—H8	118.5	C2—N1—C13	116.47 (14)
N9—C11—H11A	109.5	C6—N1—C13	121.06 (14)
N9—C11—H11B	109.5	C2—N3—C4	123.83 (14)
H11A—C11—H11B	109.5	C2—N3—C14	117.92 (15)
N9—C11—H11C	109.5	C4—N3—C14	118.23 (15)
H11A—C11—H11C	109.5	C8—N7—C6	117.17 (14)
H11B—C11—H11C	109.5	C8—N9—C11	121.75 (16)
N9—C12—H12A	109.5	C8—N9—C12	121.00 (16)
N9—C12—H12B	109.5	C11—N9—C12	117.25 (15)
H12A—C12—H12B	109.5	H1W—O1W—H2W	125 (6)
N9—C12—H12C	109.5	H3W—O2W—H4W	116 (5)
H12A—C12—H12C	109.5		
			
O1—C4—C5—C6	−176.21 (17)	N1—C2—N3—C4	0.1 (2)
N3—C4—C5—C6	4.2 (3)	O2—C2—N3—C14	−0.2 (3)
C4—C5—C6—N7	178.88 (15)	N1—C2—N3—C14	178.55 (15)
C4—C5—C6—N1	−3.3 (3)	O1—C4—N3—C2	177.81 (15)
O2—C2—N1—C6	179.72 (15)	C5—C4—N3—C2	−2.6 (2)
N3—C2—N1—C6	1.0 (2)	O1—C4—N3—C14	−0.6 (3)
O2—C2—N1—C13	−1.1 (2)	C5—C4—N3—C14	179.01 (15)
N3—C2—N1—C13	−179.84 (14)	N9—C8—N7—C6	174.40 (15)
N7—C6—N1—C2	178.70 (13)	C5—C6—N7—C8	−24.2 (3)
C5—C6—N1—C2	0.6 (2)	N1—C6—N7—C8	157.94 (14)
N7—C6—N1—C13	−0.5 (2)	N7—C8—N9—C11	−2.9 (3)
C5—C6—N1—C13	−178.56 (14)	N7—C8—N9—C12	178.23 (18)
O2—C2—N3—C4	−178.64 (15)		

### Hydrogen-bond geometry (Å, °)

**Table:**

*D*—H···*A*	*D*—H	H···*A*	*D*···*A*	*D*—H···*A*
O1W—H1W···O2	0.86 (1)	1.93 (1)	2.784 (2)	173 (3)
O1W—H2W···O1W^i^	0.86 (7)	2.01 (4)	2.771 (4)	147 (6)
O2W—H3W···O1	0.85 (1)	2.01 (2)	2.808 (2)	157 (3)
O2W—H4W···O1W^ii^	0.86 (3)	1.95 (2)	2.777 (3)	163 (6)

Symmetry codes: (i) −*x*, −*y*+1, −*z*+2; (ii) *x*, *y*, *z*−1.
